# Evaluating the Antioxidant Properties of the Leaves and Stems of *Alpinia oxyphylla* In Vitro and Its Growth-Promoting, Muscle Composition Change, and Antioxidative Stress Function on Juvenile *Litopenaeus vannamei*

**DOI:** 10.3390/antiox12101802

**Published:** 2023-09-27

**Authors:** Jun-Tao Li, Yu-Hua Zhao, Yuan Lv, Xin Su, Wen-Li Mei, Yao-Peng Lu, Pei-Hua Zheng, Ze-Long Zhang, Xiu-Xia Zhang, Hui-Qin Chen, Hao-Fu Dai, Jian-An Xian

**Affiliations:** 1 Hainan Provincial Key Laboratory for Functional Components Research and Utilization of Marine Bio-Resources, Key Laboratory of Natural Products Research and Development from Li Folk Medicine of Hainan Province, Institute of Tropical Biosciences and Biotechnology, Chinese Academy of Tropical Agricultural Science, Key Laboratory for Biology and Genetic Resources of Tropical Crops of Hainan Province, Hainan Institute of Tropical Agricultural Resources, Haikou 571101, China; lijuntao@itbb.org.cn (J.-T.L.); 13006160035@163.com (Y.L.); suxinnpc@126.com (X.S.); meiwenli@itbb.org.cn (W.-L.M.); luyaopeng@itbb.org.cn (Y.-P.L.); zhengpeihua@itbb.org.cn (P.-H.Z.); zhangzelong@itbb.org.cn (Z.-L.Z.); zhangxiuxia@itbb.org.cn (X.-X.Z.); 2 Key Lab of Freshwater Animal Breeding, Ministry of Agriculture, Key Lab of Agricultural Animal Genetics, Breeding and Reproduction of Ministry of Education, Freshwater Aquaculture Collaborative Innovation Center of Hubei Province, College of Fisheries, Huazhong Agricultural University, Wuhan 430070, China; zhaoyuhua2005@mail.hzau.edu.cn

**Keywords:** *Alpinia oxyphylla*, epicatechin, antioxidant, *Litopenaeus vannamei*

## Abstract

*Alpinia oxyphylla* is a homology of medicine and food. This study aims to investigate the dominant chemical composition and explore the antioxidant properties of the ethanol extract of the leaves and stems of *A. oxyphylla* (AOE) on juvenile shrimp, *Litopenaeus vannamei*. An in vitro test showed that AOE and its dominant chemical composition procyanidin B-2 (**1**) and epicatechin (**2**) presented DPPH and ABTS radical scavenging activities. A shrimp feeding supplement experiment revealed that shrimp growth parameters and muscle composition were improved significantly when fed with a 200 mg/Kg AOE additive. Meanwhile, the activities of antioxidant enzymes (CAT, GSH-Px, SOD, and T-AOC) in serum and the liver and the expression of related genes (*LvMn-SOD*, *LvCAT*, *LvproPo*, and *LvGSH-Px*) were enhanced with various degrees in different AOE additive groups while the content of MDA was significantly decreased. Moreover, the antioxidative effect of AOE additive groups on shrimp was also observed in an acute ammonia nitrogen stress test.

## 1. Introduction

Oxidative stress is one of pivotal factors that cause disease and aging in organism [1,2]. It occurs when a reactive oxygen species (ROS) is overproduced and loses the balance between ROS production and availability by antioxidant defensive system. Accumulation of ROS can cause cell and tissue damage by impairing membrane, enzymes, and DNA [3]. Flavonoid and phenolic compound-rich foods, such as onions [4], buchwheat [5], grape seeds [6], etc., are always welcomed in daily life due to their antioxidant function. They are able to alleviate oxidative stress by scavenging ROS and/or enhancing the activities of glutathione peroxidase (GSH-Px), superoxide dismutase (SOD), catalase (CAT), and other antioxidant enzymes or substances [7]. Currently, antioxidants are not only used in food additives, health care products, and cosmetics, but also in feed supplements and stabilizers in the industry.

*Litopenaeus vannamei*, one of the most popular aquatic commercial shrimp, also named the white leg shrimp or vannamei shrimp, contains very high crude protein; therefore, the production has notably increased in recent years [8]. However, *L. vannamei* is very sensitive to the environment due to the high density culture, and the changes in temperature, dissolved oxygen, ammonia nitrogen, nitrite, and microorganism may lead to the shortage of oxygen, which would induce oxidative stress and cause various diseases [9,10]. Antioxidants, such as vitamin C, have reportedly been employed as feed additives to alleviate oxidative stress caused by heavy metal toxicity and pesticides [11]. Currently, environmental and health friendly herbs with antioxidant activity have been more and more welcomed by fish farmers to improve the growth, immune system, and the antioxidant capacity of *L. vannamei*. 

*Alpinia oxyphylla* Miq. (Zingiberaceae), known as one of the “four Southern Herban Medicines” in China, is also used as vegetables and preserved fruits, with traditional effects of anti-diuretic and anti-diarrheal as well as neuroprotective activity [12]. Pharmacological studies have reported that *A. oxyphylla* has various functions, such as memory improvement, anti-oxidation, anti-aging, etc. [13,14]. However, the esculent part of *A. oxyphylla* is the fruits, and the leaves and stems are usually discarded as waste after harvesting the fruits for 3–4 years. Previous studies showed that an abundance of phenolic compounds contained in different tissues of *A. oxyphylla*, mainly consisted of flavonoids, phenolic acids, and tannins, which usually present potential antioxidant activity [15,16,17].

In this study, the antioxidant activity of the extract of leaves and stems of *A. oxyphylla* and its main chemical constituents were evaluated by 2,2-diphenyl-1-picryhydrazyl radical (DPPH) and 2,2′-azino-bis(3-ethylbenzo-thiazoline-6-sulfonic acid radical (ABTS) scavenging activity assay. Meanwhile, the extract was used as a feed additive of *L. vannamei*, and in vivo, an antioxidative ability assessment was carried out by examining the enzyme activities (CAT, GSH-Px, SOD, T-AOC, and MDA), and antioxidant related genes expression as well as the ability of ammonia nitrogen stress resistance. The results of this study may provide important evidence for the utilization of the discarded part of *A. oxyphylla* and, meanwhile, explore a new option of functional feed additive of aquaculture.

## 2. Materials and Method

### 2.1. Materials

A total of 4000 healthy and disease-free shrimp (*Litopenaeus vannamei*) were obtained from Wenchang Scientific Research and Test Base, Institute of Tropical Biosciences and Biotechnology, Chinese Academy of Tropical Agricultural Science. First, shrimp were fed on a basal diet twice daily for two weeks, and then, 540 of them with high vitality and initial body weight of 2.07 ± 0.02 g were selected as experimental materials.

### 2.2. Plant Sample Extraction and Experimental Diets Preparation

The fresh leaves and stems of *A. oxyphylla* were collected from Wenchang in Hainan of China in June 2020, cut into 1–2 cm pieces, and extracted with 95% ethanol by heating reflux three times (2 h each time). Subsequently, the ethanol extract was filtered, and then, the filtrate was concentrated by rotary evaporator under reduced pressure. The *A. oxyphylla* extract (AOE) was obtained and stored at −20 °C for further use.

### 2.3. Experimental Diets Preparation

*L. vannamei* No. 1 diet as the basal diet was purchased from Hainan Yuehai Co., Ltd. (Hainan, China), pounded into powder, and then passed through 80 mesh sieve. Afterwards, the diets with a serials of different AOE concentrations (0, 100, 200, 300, 500, and 700 mg/Kg) were prepared.

### 2.4. Main Chemical Constituents Isolation and Identification

#### 2.4.1. LC-MS Analysis of AOE

The chemical constituents of AOE were analyzed using Thermo UltiMate 3000 ultra-high pressure liquid chromatography (UHPLC) system coupled with Bruker Compact mass spectrometry (UHPLC-ESI-MS). The separation was performed on a COSIMOSIL π-NAP packed column (4.6 × 250 mm, 5 μm, Cosmosil, Japan). The acidified ultrapure water (0.1% formic acid, solvent A) and methanol (solvent B) were used as mobile phases, and the flow rate of 0.4 mL/min was set. The gradient condition was performed as follows: 10% B (0–5 min), 10–45% B (5–125 min), 45–100% B (125–135 min), 100% B (135–140 min).

#### 2.4.2. Main Chemical Constituents Isolation and Identification

According to LC-MS analysis result, there were two dominant peaks in AOE appeared at *t*_R_ = 72.3 min and *t*_R_ = 83.3 min, respectively. Therefore, the isolation of these two compounds were carried out by various chromatographic techniques.

AOE (5.2 g) was applied to ODS gel (20–45 μM, Fuji Silysia Chemical Co., Ltd., Greenville, NC, USA) column eluting with MeOH/H_2_O (*v*/*v*, 1:9, 2:8, 3:7, 4:6, 5:5, 9:1, 1:0, each 400 mL) to obtain 10 fractions (Frs. 1–10). By analysis of the fractions, the two target compounds were contained in Fr. 3 and Fr. 6, respectively. Thereby, Fr. 3 was submitted to semi-preparative HPLC performed with an Agilent Technologies 1260 Infinity II equipped with an Agilent DAD G1315D detector (Agilent, CA, USA) (COSMOSIL π-NAP packed column, 10 × 250 mm, 5 μm, Cosmosil, Kyoto, Japan; 38% MeOH/H_2_O, *v*/*v*; flow rate 4.0 mL/min; UV detection at 210/280 nm) to get compound **1**. Fr. 6 was repeatedly subjected to semi-preparative HPLC (π-NAP column; 42% MeOH/H_2_O, *v*/*v*; flow rate 4.0 mL/min; UV detection at 210/280 nm) to acquire compound **2**.

The isolated compounds were sent to Bruker AVIII NMR spectrometer (Bruker, Baden-Wurttemberg, Germany) to get ^1^H (500 MHz) and ^13^C NMR (125 MHz) spectra. Chemical shifts were referenced to the solvent residual peaks.

### 2.5. Antioxidant Activities Assay of AOE and the Main Compounds In Vitro

#### 2.5.1. DPPH Radical Scavenging Activity Assay

The DPPH (purity ≥ 98.5, Sigma-Aldrich Co., Ltd., St. Louis, MO, USA) scavenging activity was conducted as reference described [18]. Briefly, 320 μL different concentrations of sample in DMSO and 320 μL DPPH reagent (0.1 mM) were mixed in 1.5 mL centrifuge tube and reacted avoiding light for 30 min, and then, 200 μL were take out and added to 96-well plate for measuring the absorbance with a microplate reader at 517 nm (OD_sample_). The absorbance of the mixture solution of sample in DMSO and ethanol was set as control (OD_control_) while the absorbance of the mixture solution of DMSO and DPPH reagent was set as blank (OD_blank_). Vitamin C was the positive control. The DPPH radical scavenging capacity was calculated using the following formula:

DPPH radical scavenging capacity (%) = [1 − (OD_sample_ − OD_control_)/OD_blank_] × 100%

#### 2.5.2. ABTS Radical Scavenging Activity Assay

The ABTS (purity ≥ 98, Sigma-Aldrich Co., Ltd., MO, USA) radical scavenging activity was evaluated as previously reported [18,19]. Briefly, 160 μL different concentrations of sample in DMSO and 480 μL ABTS reagent were mixed in 1.5 mL centrifuge tube and reacted avoiding light for 6 min, and then, 200 μL was taken out and added to 96-well plate for measuring the absorbance with a microplate reader at 734 nm (OD_sample_). The absorbance of the mixture solution of sample in DMSO and water was set as control (OD_control_) while the absorbance of the mixture solution of DMSO and ABTS reagent was set as blank (OD_blank_). Trolox was the positive control. The ABTS radical scavenging capacity was calculated using the following formula:

ABTS radical scavenging capacity (%) = [1 − (OD_sample_ − OD_control_)/OD_blank_] × 100%

### 2.6. L. vannamei Feeding Supplement Experiment

An amount of 540 shrimp were randomly divided into six groups, with three replicates per groups (30 shrimp per replicate). Shrimp in each replicate were reared in 79 cm × 58 cm × 66 cm cage with sea water: fresh water = 3:1, and they were fed at 6:00, 10:00, 14:00, and 18:00 o’ clock every day. The daily feeding amount was 2–6% of the body weight. The experiment lasted for 8 weeks.

The residual diet and feces were siphoned off after feeding for 1 h, followed by backing dry and recording their weight. The water temperature, pH, dissolved oxygen, and salinity were maintained at 30 ± 2 °C, 8.0 ± 0.3, 6.0 mg/L, and 1.0–1.5%, respectively.

### 2.7. Acute Ammonia Nitrogen Stress Test

After the feeding trial, randomly, 15 healthy shrimp were collected from each group for acute ammonia nitrogen stress test after fasting for 24 h. The stress test used 100 mg/L ammonia chloride, and the test time was 24 h.

### 2.8. Shrimp Samples Collection

Randomly, 6 shrimp were collected from each replicate of the control and experimental groups after fasting for 24 h. First, the body weight and body length of each shrimp were recorded. Hemolymph was collected from the cardiocoelom, followed by timely refrigerated centrifugation at 3500 rpm for 10 min, and the supernatant as plasma was rapidly frozen in liquid nitrogen. Subsequently, the liver and muscle were obtained and frozen in liquid nitrogen as well. All the samples were stored at −80 °C for further use after collection.

### 2.9. Growth Performance

The weight gain rate (WGR), length gain rate (LGR), special growth ratio (SGR), survival ratio (SR), and feed efficiency ratio (FER) were calculated as follows [20]:

Weight gain rate (WGR, %) = (final body weight − initial body weight)/initial body weight × 100%

Length gain rate (LGR, %) = (final body length − initial body length)/initial body length × 100%

Specific growth rate (SGR, %/day) = [Ln(final body weight) − Ln(initial body weight)]/duration of experiment × 100%

Survival rate (SR, %) = final number/initial number × 100%

Feed efficiency rate (FER, %) = (final body weight − initial body weight)/dried diet consumption

### 2.10. Proximate Composition

Moisture, crude ash, crude protein, and crude lipid of basal diet as well as whole shrimp, muscle, and liver were evaluated with the methods of the Association of Official Analytical Chemists [21].

### 2.11. Antioxidative Stress Function In Vivo

#### 2.11.1. Enzymes Activity Assay

The activities of SOD, CAT, GSH-Px, and MDA content as well as T-AOC in plasma and liver were analyzed using the respective kits (Nanjing Jiancheng Bioengineering Institute, Nanjing, China).

#### 2.11.2. RNA Extraction and Gene Expression

Total RNA extraction from shrimp was carried out with animal total RNA isolation Kit (Foregene, Chengdu, China). RNA quality was detected by agarose gel electrophoresis while the purity and concentration of RNA were tested using microUV–visible spectrophotometer with absorbance ratio at 260/280 nm. The RNA with its absorption value between 1.8–2.2 was used as the reverse transcription template. Subsequently, genomic DNA was removed, and cDNA was synthesized using Prime ScriptTM RT reagent Kit. Then, primers were designed by Primer 5.0 based on cDNA sequence of *L. vannamei* from NCBI database as shown in Table S1 and synthesized by Shanghai Sangon Biotech Co., Ltd. (Shanghai, China).

Quantitative real-time PCR was conducted using SYBR^®^ Green qPCR Mix (Guangzhou Dongsheng Biotechnology Co., Ltd., Guangzhou, China). β-actin was used as the reference gene, and the relative expressions of the target genes were calculated using the 2^−ΔΔCt^ method.

### 2.12. Statistical Analysis

All the data were presented as means ± standard deviation (SD), and SPSS18.0 software (SPSS Inc., Chicago, IL, USA) was used for statistical analyses. One-way analysis of variance and Duncan were used to assess the differences among groups. A value of *p* < 0.05 was considered statistical significant difference between groups.

## 3. Results and Discussion

### 3.1. Main Chemical Constituents Identification

**Figure 1:**
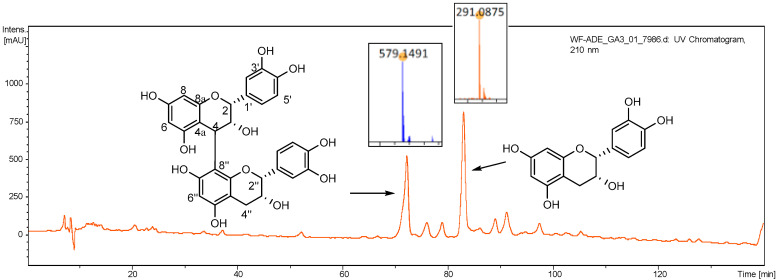
HPLC-UV spectrum (λ_210_ nm) of AOE and the structures of identified main compounds.

Two major chemical constituents in AOE were identified as procyanidin B-2 (**1**) and epicatechin (**2**) (Figure 1), respectively, by comparison of the MS and NMR data with those reported by references [22,23]. Both compounds are flavane, and (**1**) is structurally the dimer of (**2**).

Procyanidin B-2 (**1**): Brown gum; ESI-MS *m*/*z* 579.1491 [M+H]^+^; ^1^H NMR (500 MHz, MeOD) *δ*_H_: 5.07 (1H, br s, H-2), 4.22 (1H, br s, H-3), 4.65 (1H, br s, H-4), 6.01 (1H, br s, H-6), 5.94 (1H, br s, H-8), 6.96 (1H, br s, H-2′), 6.77 (4H, m, overlapped, H-5′, 6′, 5‴, 5‴), 5.01 (1H, br s, H-2″), 4.26 (1H, br s, H-3″), 2.85 (1H, br d, *J* = 16.4 Hz, H-4″a), 2.95 (1H, br d, *J* = 16.4 Hz, H-4″b), 7.06 (1H, br s, H-2‴); ^13^C NMR (125 MHz, MeOD) *δ*_C_: 77.3 (C-2), 73.0 (C-3), 36.8 (C-4), 100.4 (C-4a), 158.6 (C-5), 95.9 (C-6), 157.8 (C-7), 96.2 (C-8), 156.2 (C-8a), 132.6 (C-1′), 115.3 (C-2′), 145.6 (C-3′), 145.9 (C-4′), 115.9 (C-5′), 119.4 (C-6′), 79.7 (C-2″), 67.4 (C-3″), 29.7 (C-4″), 100.5 (C-4″a), 158.8 (C-5″), 97.2 (C-6″), 157.8 (C-7″), 107.8 (C-8″), 156.2 (C-8″a), 132.6 (C-1‴), 115.9 (C-2‴), 145.5 (C-3‴), 145.9 (C-4‴), 115.3 (C-5‴), 119.4 (C-6‴).

Epicatechin (**2**): Yellow crystal; ESI-MS *m*/*z* 291.0875 [M+H]^+^; ^1^H NMR (500 MHz, MeOD) *δ*_H_: 4.81 (1H, br. s, H-2), 4.17 (1H, m, H-3), 2.73 (1H, dd, *J* = 16.7, 2.7 Hz, H-4a), 2.86 (1H, dd, *J* = 16.7, 4.6 Hz, H-4b), 5.91 (1H, d, *J* = 2.0 Hz, H-6), 5.94 (1H, d, *J* = 2.0 Hz, H-8), 6.97 (1H, d, *J* = 2.0 Hz, H-2′), 6.76 (1H, d, *J* = 8.3 Hz, H-5′), 6.80 (1H, dd, *J* = 8.3, 2.0 Hz, H-6′); ^13^C NMR (125 MHz, MeOD) *δ*_C_: 79.8 (C-2), 67.5 (C-3), 29.5 (C-4), 157.6 (C-5), 96.4 (C-6), 157.3 (C-7), 95.9 (C-8), 158.0 (C-9), 100.1 (C-10), 132.3 (C-1′), 115.9 (C-2′), 145.7 (C-3′), 145.9 (C-4′), 115.3 (C-5′), 119.4 (C-6′).

### 3.2. Antioxidant Activities of AOE and Its Main Chemical Constituents In Vitro

As shown in Table 1, AOE presented weak DPPH radical scavenging activity with the inhibitory rate of 58.59 ± 0.52% at the concentration of 200 μg/mL (vitamin C as the positive control, with the inhibitory rate of 85.44 ± 0.34%) while it showed strong ABTS radical scavenging activity with the inhibitory rate of 92.78 ± 0.43% at the same concentration, almost the same as the positive control (trolox) that had an inhibitory rate of 92.69 ± 0.03%.

As the dominant chemical constituents, procyanidin B-2 (**1**) and epicatechin (**2**) presented significant antioxidant activity by DPPH and ABTS radical scavenging activities, with IC_50_ values of 12.00 ± 0.12 μg/mL and 14.72 ± 0.25 μg/mL for **1** while 2.53 ± 0.15 μg/mL and 2.53 ± 0.03 μg/mL for **2**, respectively (IC_50_ of vitamin C was 6.20 ± 0.41 μg/mL, and IC_50_ of trolox was 31.61 ± 0.60 μg/mL).

By DPPH and ABTS radical scavenging activities, both AOE and its major components showed antioxidant activities in vitro by eliminating free radicals. Epicatechin (**2**) could be regarded as the basic structure possessing antioxidant activity. The epicatechin unit presented significant effects due to the structural features of ortho-dihydroxy at B ring and hydroxyl groups at positions 3 and 5, which are helpful for electron delocalization and provide hydrogen bonding with the oxo group [24]. Moreover, epicatechin (**2**) has been reported as a potent natural antioxidant [25] with hepatocyte protective [26], cardiovascular protective [27], and anti-aging effects [28] previously. Therefore, epicatechin (**2**) may be one of the important antioxidative chemical compositions in AOE.

### 3.3. The Functions of AOE as Additive in Shrimp

#### 3.3.1. Growth Performance and Survival Rate

The effects of AOE on growth performance of shrimp after eight weeks of the feeding experiment are shown in Table 2. From the results, the groups with 100 and 200 mg/Kg AOE additives obtained an obvious increase in LGR, WGR, SGR, and FER compared to the control group (*p* < 0.05); the group with a 300 mg/Kg AOE additive significantly increased in LGR, WGR, and SGR (*p* < 0.05); and the groups with 500 and 700 mg/Kg AOE additives presented no remarkable difference with control group (*p* > 0.05). Based on the data, the growth performance indexes of shrimp with AOE additive groups generally appeared better than the control group, whereas the effect of AOE additives on the LGR, WGR, SGR, and FER of shrimp increased first and then decreased with the content increasing. The group with a 200 mg/Kg AOE additive (AOE-2) presented the highest values for all the indexes. Nevertheless, no significant difference in SR between the experimental and control groups was observed (*p* > 0.05).

Medicinal plants as feed additives have frequently received unexpected results in animals due to their functions of promoting growing by increasing the diet absorption capacity [29], improving immunity by anti-inflammatory activities and detoxifying the body [30], and increasing the reproductive rate by promoting egg production and excretion [31]. In this study, AOE additive showed the effect on the growth-promoting of shrimp, and 200 mg/Kg was presented as the desirable content. No difference in SR means no toxicity of the plant sample.

#### 3.3.2. Muscle Proximate Composition

Muscle proximate composition of *L. vannamei* is shown in Table 3. The results showed that moisture and ash were a minor difference between the AOE additive groups and control group (*p* > 0.05). However, with the increase in AOE additive content, the content of crude protein increased first and then decreased while the concentration variation of crude lipid was opposite. When the additive content was 200 mg/Kg, the content of crude protein and crude lipid reached their peaks at 90.57 ± 1.13% and 8.37 ± 0.07%, respectively, and presented a significant difference with the control group (*p* < 0.05).

High protein content is one of the evaluation factors of high quality products. Previous research has proved that medicinal plants are able to change the ingredient of animal bodies and muscles. For example, oregano essential oil can enhance muscle protein sedimentation in channel catfish [32], and *Scutellaria baicalensis* can improve the ratio of crude protein and crude lipid of rabbit fish [33]. Herein, 200 mg/Kg AOE would be a good choice to improve the quality of shrimp by changing the ratio of crude protein and crude lipid in the muscle.

#### 3.3.3. Effects of AOE on Antioxidant Activity in *L. vannamei*

Shrimp defense pathogens, when attacking, mainly rely on nonspecific immunity; therefore, strengthening the immunity is essential to raise the survival rate in an aquaculture. It is evident that boosting antioxidant capacity is an effective way to improve immunity, which is reflected by the activities of antioxidant-related enzymes or substances, including CAT, GSH-Px, and SOD as well as the content of MDA [34]. T-AOC is an index to reflect the total antioxidative level of an organism.

##### AOE Additive Affected on Activities of CAT, GSH-Px, SOD, and T-AOC as well as the Content of MDA in Shrimp

**Figure 2:**
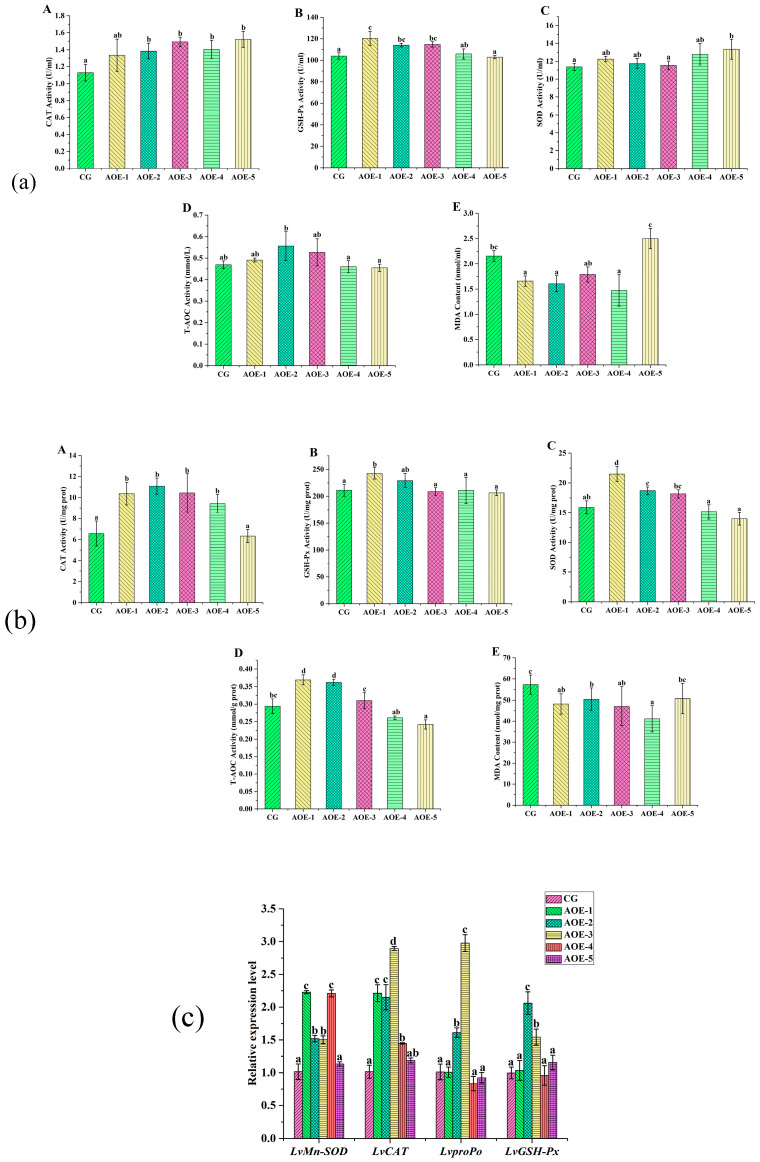
Antioxidant enzyme activity assay and related genes expression in shrimp fed with different concentration of AOE additive for 8 weeks. (**a**) Antioxidant enzyme activity assay in plasma of shrimp; (**b**) Antioxidant enzyme activity assay in liver of shrimp; (**c**) Antioxidant gene expression in liver of shrimp. (**A**): CAT activity; (**B**): GSH-Px activity; (**C**): SOD activity; (**D**): T-AOC; and (**E**): MDA content. (n = 9).

After eight weeks of the feeding trial, the levels of the activities of CAT, GSH-Px, SOD, and T-AO, and the content of MDA in plasma of shrimp are shown in Figure 2a. When AOE additive concentration ≥ 200 mg/Kg (AOE-2, 3, 4, 5), the activity of CAT was significantly enhanced compared to the control group (*p* < 0.05) while the activity of GSH-Px presented remarkably enhanced in groups AOE-1, AOE-2, and AOE-3 (*p* < 0.05). Meanwhile, the groups with 700 mg/Kg (AOE-5) and 200 mg/Kg (AOE-2) of AOE concentration could increase the activities of SOD and T-AOC, respectively (*p* < 0.05). Moreover, a dramatic decrease in the content of MDA was observed in groups AOE-1, AOE-2, and AOE-4. Based on above, 200 mg/Kg of an AOE (AOE-2) additive to shrimp could significantly increase the activities of antioxidant enzymes in shrimp.

In the liver, the levels of CAT, GSH-Px, SOD, and T-AOC activities showed similar variation trends that increased first and then decreased with an increasing concentration of an AOE additive (Figure 2b). AOE-1 group had the highest levels for GSH-Px, SOD, and T-AOC activities while AOE-2 group had the highest level for CAT activity. Significant improvement of CAT, GSH-Px, SOD, and T-AOC activities could be found in AOE-1 and AOE-2 groups (*p* < 0.05). The content of MDA in the liver decreased in AOE additive groups compared to the control group, and a significant decrease was observed in groups AOE-1–4 (*p* < 0.05).

##### AOE Additive Effect on the Levels of Antioxidant-Related Genes Expression in Liver of *L. vannamei*

Relative expression levels of antioxidant-related genes in the liver of *L. vannamei* influenced by an AOE additive are shown in Figure 2c. Compared to the control group, the expressions of *LvMn-SOD* and *LvCAT* were significantly increased in the groups AOE-1–4 (*p* < 0.05). The gene expression of *LvMn-SOD* in AOE-1–4 were 2.23, 1.52, 1.50, and 2.21 times that of the control, respectively. Meanwhile, the gene expression of *LvCAT* in the same groups were 2.55, 2.15, 2.89, and 1.45 times increased, respectively.

Moreover, the expressions of *LvproPo* and *LvGSH-Px* were significantly increased in the groups of AOE-2 and AOE-3 (*p* < 0.05). *LvproPo* expression in the above two groups were 1.61 and 2.98 times that compared with the control, and likewise, the expression of *LvGSH-Px* were 2.06 and 1.54 times those for the same groups, respectively.

Flavonoid-rich medicinal plants as additives to aquatic animals are beneficial to enhance antioxidant function [35,36] because flavonoids not only can eliminate free radicals directly, but also can act as antioxidants by enhancing the activities of antioxidant-related enzymes, which are conducted by inducing the expression of the electrophile responsive element (EpRE)-mediated gene due to their redox properties [24].

#### 3.3.4. Effects of AOE on Antioxidant Activity in *L. vannamei* after Acute Ammonia Nitrogen Stress Test

Ammonia nitrogen is an unavoidable pollution stress in aquacultures [37]. The accumulation of ammonia nitrogen in aquatic animals can cause metabolic disorders, growth problems, and even death due to the damage of the antioxidant system and immune system [38]. Boosting antioxidant activity has the great significance to resist ammonia nitrogen stress.

##### AOE Additive Effects on Activities of CAT, GSH-Px, SOD, and T-AOC as well as the Content of MDA in Shrimp after Acute Ammonia Nitrogen Stress Test

**Figure 3:**
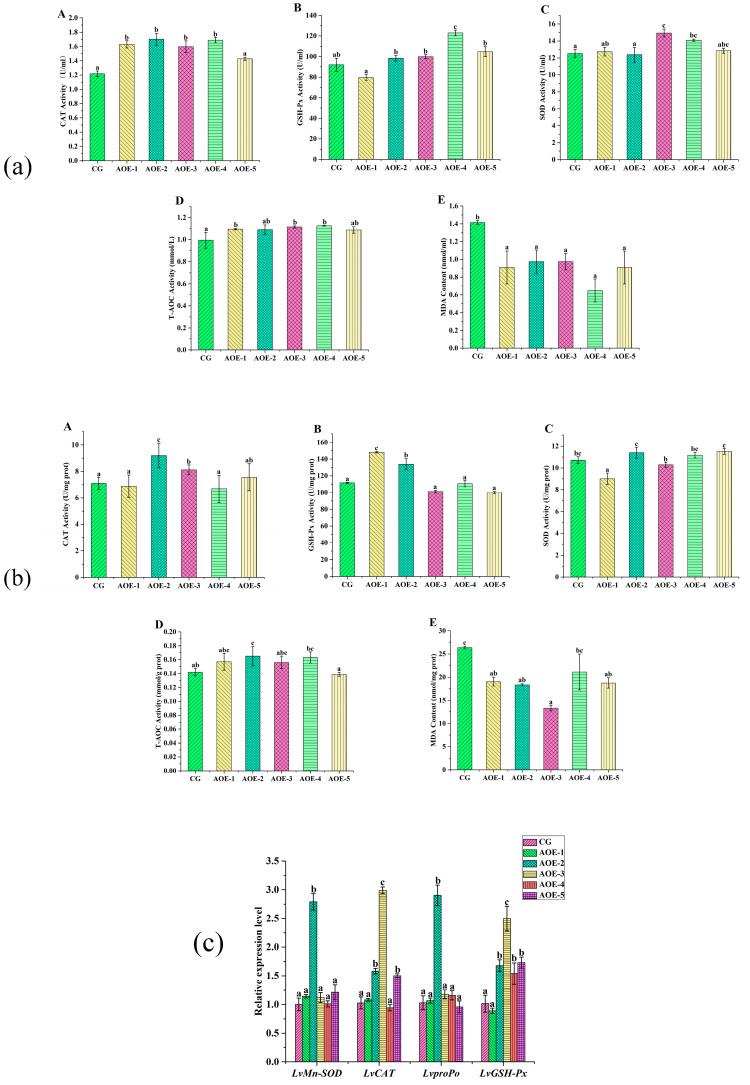
Antioxidant enzyme activity assay and related gene expression in shrimp fed with different concentrations of AOE additive after acute ammonia nitrogen stress test. (**a**) Antioxidant enzyme activity assay in plasma of shrimp; (**b**) Antioxidant enzyme activity assay in liver of shrimp; (**c**) Antioxidant gene expression in liver of shrimp. (**A**): CAT activity; (**B**): GSH-Px activity; (**C**): SOD activity; (**D**): T-AOC; and (**E**): MDA content. (n = 9).

After the acute ammonia nitrogen stress test, AOE additive groups enhanced the activities of CAT, GSH-Px, SOD, and T-AOC in shrimp on different levels (Figure 3). In plasma (Figure 3a), the AOE-4 group significantly increased the activities of more than four enzymes (*p* < 0.05), whereas AOE-3 obviously enhanced the activities of CAT, SOD, and T-AOC (*p* < 0.05). All the AOE additive groups dramatically decreased the levels of MDA content in plasma (*p* < 0.05).

In liver (Figure 3b), the AOE-2 group was observed to enhance the activities of CAT, GSH-Px, and T-AOC dramatically (*p* < 0.05). However, no significant variation was detected for SOD (*p* > 0.05). Furthermore, all the AOE additive groups showed a decrease in MDA content, and the AOE-3 group had the lowest level (*p* < 0.05).

The above data suggested that AOE additive were able to enhance the activities of CAT, GSH-Px, SOD, and T-AOC and, meanwhile, decrease the content of MDA, which was helpful for shrimp to resist high ammonia nitrogen content induced by oxidative stress.

##### AOE Additive Effect on the Levels of Antioxidant Related Genes Expression in Liver of *L. vannamei* after Acute Ammonia Nitrogen Stress Test

After the acute ammonia nitrogen stress test, the expression of antioxidant-related genes in the liver are shown in Figure 3c. It distinctly displayed that AOE-2 significantly increased the expression of all the selected antioxidant genes (*p* < 0.05), with the highest increase observed in *LvproPo* with 2.8 times that of the control. In addition, groups of AOE-3 and AOE-5 obviously increased the expression of *LvCAT* (*p* < 0.05) while the experiment groups, except for AOE-1, had a distinct effect on increasing *LvGSH-Px* expression (*p* < 0.05).

Hence, 200 mg/Kg AOE (AOE-2) could effectively increase the expression of the selected antioxidant genes either before or after the acute ammonia nitrogen stress test. From the results, it presented that the groups that increased the expression of the antioxidant-related genes were not always the same groups that enhanced the activities of the corresponding enzymes. In vivo, besides gene expression, enzyme activities can be affected by many factors, including inhibitors, stimulators, temperature, pH, etc. [39]. Herein, AOE could directly affect the activities of CAT, GSH-Px, SOD, and T-AOC as well as enhance the gene expression.

CAT, GSH-Px, SOD, T-AOC, and MDA are important indicators to evaluate the antioxidant enzyme defense system. SOD, GSH-Px, and CAT work synergistically to remove ROS. SOD catalyses the dismutation of O_2_^−^ to form H_2_O_2_, and GSH-Px and CAT are responsible for removing H_2_O_2_ from the cellular environment [40]. The T-AOC value presents the total enzymatic and non-enzymatic antioxidant capacity. MDA is the production of lipid peroxidation and can lead to cell damage by attaching with DNA, lipids, and proteins [41]. In this study, AOE additive groups improved the antioxidant ability of juvenile *L. vannamei* by enhancing the activities of CAT, GSH-Px, SOD, T-AOC and the expressing of antioxidant-related genes as well as reducing the content of MAD.

## 4. Conclusions

In this study, the main compounds were isolated from AOE guided by LC-MS and identified as procyanidin B-2 (**1**) and epicatechin (**2**). DPPH and ABTS radical scavenging ability were used to evaluate the antioxidant activity in vitro, and both AOE and its dominant chemical constituents presented significant activity. The IC_50_ values of procyanidin B-2 (**1**) were 12.00 ± 0.12 μg/mL and 14.75 ± 0.25 μg/mL, respectively, for DPPH (IC_50_ value of 6.20 ± 0.41 μg/mL for vitamin C) and ABTS (IC_50_ value of 31.61 ± 0.60 μg/mL for trolox) radical scavenging assay while 2.53 ± 0.15 and 2.53 ± 0.03 μg/mL for epicatechin (**2**). AOE supplement experiment was carried out on *L. vannamei*, and the improvement of shrimp growth parameters (WGR, LGR, SGR, SR, FER) was observed in AOE supplement groups as well as crude protein content increase and crude lipid content decrease in the muscle. The effects of AOE supplement on growth parameters increased first and then decreased with the concentration increasing, and the group with 200 mg/Kg AOE additive presented the highest values for all the indexes. The activities of antioxidant-related enzymes (CAT, GSH-Px, SOD, and T-AOC) in the liver and serum varied at different AOE supplement groups. Among them, the group with 100 mg/Kg (AOE-1) significantly increased the activities of CAT, GSH-Px, SOD, and T-AOC and decreased the content of MDA. Groups AOE-1 and AOE-2 greatly improved the expression of antioxidant-related genes (*LvMn-SOD*, *LvCAT*, *LvproPo*, and *LvGSH-Px*) in the liver. Furthermore, the acute ammonia nitrogen stress test results showed that AOE-2 group notably increased the activities of CAT, GSH-Px, SOD, and T-AOC and decreased the content of MDA as well as enhanced the expression of *LvMn-SOD*, *LvCAT*, *LvproPo*, and *LvGSH-Px*. Based on the above, the discarded part of *A. oxyphylla* could be developed into additives to relieve oxidative stress in aquatic animals, and in the flavonoids especially, the dominant compounds procyanidin B-2 and epicatechin played a pivotal role.

## Figures and Tables

**Table 1:** Antioxidant activities of AOE and the main chemical composition analyzed with different methods.

	DPPH Radical Scavenging Activity		ABTS Radical Scavenging Activity	
	Inhibitory Rate (%) ^a^	IC_50_ (μg/mL)	Inhibitory Rate (%) ^a^	IC_50_ (μg/mL)
AOE	58.59 ± 0.52 *		92.78 ± 0.43	
1	50.12 ± 0.01 *	12.00 ± 0.12 *	90.44 ± 0.01 *	14.72 ± 0.25 *
2	80.69 ± 1.24	2.53 ± 0.15 *	92.39 ± 0.27	2.53 ± 0.03 *
vitamin C ^b^	85.44 ± 0.34	6.20 ± 0.41		
trolox ^b^			92.69 ± 0.03	31.61 ± 0.60

^a^ at the concentration of 200 μg/mL. ^b^ positive control. n = 3, and asterisks indicated significant difference from the positive control (*p* < 0.05).

**Table 2:** Growth performance of shrimp fed on diets containing different concentrations of AOE for 8 weeks.

	0	100 mg/Kg	200 mg/Kg	300 mg/Kg	500 mg/Kg	700 mg/Kg
Initial body weight, IW (g)	2.06 ± 0.01	2.05 ± 0.02	2.04 ± 0.01	2.09 ± 0.01	2.09 ± 0.01	2.08 ± 0.00
Final body weight, FW (g)	4.02 ± 0.11	4.35 ± 0.48	4.29 ± 0.16	4.37 ± 0.28	4.04 ± 0.38	4.25 ± 0.25
Weight gain rate, WGR (%)	95.30 ± 3.00 ^a^	114.81 ± 13.52 ^b^	117.73 ± 5.77 ^b^	114.03 ± 12.93 ^b^	103.00 ± 2.14 ^ab^	98.24 ± 18.46 ^a^
Initial body length, IL (cm)	4.98 ± 0.16	4.88 ± 0.14	4.86 ± 0.07	4.91 ± 0.12	4.98 ± 0.11	4.94 ± 0.11
Final body length, FL (cm)	8.38 ± 0.92	9.12 ± 0.67	8.94 ± 0.76	9.14 ± 0.67	8.86 ± 0.84	8.55 ± 0.91
Length gain rate, LGR (%)	74.76 ± 10.08 ^a^	80.27 ± 7.09 ^b^	84.90 ± 3.85 ^b^	84.54 ± 12.73 ^b^	79.95 ± 4.59 ^b^	74.09 ± 3.33 ^a^
Specific growth rate, SGR (%)	1.20 ± 0.01 ^a^	1.37 ± 0.05 ^b^	1.39 ± 0.00 ^b^	1.36 ± 0.05 ^b^	1.27 ± 0.01 ^ab^	1.22 ± 0.08 ^a^
Survival rate, SR (%)	77.78 ± 5.09	80.00 ± 6.67	81.11 ± 5.09	75.56 ± 11.71	81.11 ± 1.92	78.89 ± 3.85
Feed efficiency ration, FER (%)	40.48 ± 5.83 ^a^	47.81 ± 2.70 ^b^	48.30 ± 1.17 ^b^	46.47 ± 3.32 ^ab^	43.95 ± 0.80 ^ab^	41.24 ± 3.13 ^a^

The different superscript values represent a significant difference (*p* < 0.05) in the same row.

**Table 3:** Effects of AOE additive on shrimp muscle composition fed on diets containing different concentrations of AOE for 8 weeks.

	Moisture, %	Ash, %	Crude Protein, %	Crude Lipid, %
0 mg/Kg	77.46 ± 0.26	6.50 ± 0.04	88.06 ± 0.34 ^a^	13.88 ± 0.15 ^b^
100 mg/Kg	76.85 ± 0.21	6.29 ± 0.11	88.85 ± 0.85 ^ab^	11.05 ± 0.16 ^ab^
200 mg/Kg	76.81 ± 0.23	6.23 ± 0.06	90.57 ± 1.13 ^b^	8.37 ± 0.07 ^a^
300 mg/Kg	77.04 ± 0.18	6.38 ± 0.11	89.90 ± 0.61 ^ab^	13.27 ± 1.74 ^b^
500 mg/Kg	77.34 ± 0.38	6.46 ± 0.01	88.41 ± 1.05 ^ab^	14.44 ± 0.07 ^b^
700 mg/Kg	77.20 ± 0.13	6.46 ± 0.00	88.51 ± 0.60 ^ab^	13.53 ± 0.18 ^b^

The different superscript values represent a significant difference (*p* < 0.05) in the same row.

## Data Availability

All of the data is included in the article/supplementary material.

## Supplementary Materials

The following supporting information can be downloaded at: https://www.mdpi.com/article/10.3390/antiox12101802/s1, Table S1: Primer information of Real-time Fluorescence Quantitative PCR; Figure S1–S6: ^1^H and ^13^C NMR, and HEESIMS spectra of compounds **1** and **2**.

## Abbreviations

2,2-diphenyl-1-picryhydrazyl (DPPH); 2,2’-azino-bis(3-ethylbenzo-thiazoline-6-sulfonic acid (ABTS); glutathione peroxidase (GSH-Px); superoxide dismutase (SOD); catalase (CAT); total antioxidant capacity (T-AOC); malondialdehyde (MDA); reactive oxygen species (ROS); methyl sulfoxide (DMSO); optical density (OD); octadecylsilyl (ODS); methanol (MeOH); water (H_2_O); volume (v); diode array detector (DAD); hydrogen ion concentration (pH); weight gain rate (WGR); length gain rate (LGR); special growth ratio (SGR); survival ratio (SR); feed efficiency ratio (FER); ribonucleic acid (RNA); deoxyribonucleic acid (DNA); complementary deoxyribonucleic acid (cDNA); national center for biotechnology information (NCBI); polymerase chain reaction (PCR); standard deviation (SD); the half maximal inhibitory concentration (IC_50_); et cetera (etc.); high pressure liquid chromatography (HPLC); mass spectrometry (MS); electrospray ionization (ESI); ultraviolet (UV); proton nuclear magnetic resonance spectroscopy (^1^H NMR); carbon nuclear magnetic resonance spectroscopy (^13^C NMR).
