# Supplementary material for: Evaluating the Antioxidant Properties of the Leaves and Stems of Alpinia oxyphylla In Vitro and Its Growth-Promoting, Muscle Composition Change, and Antioxidative Stress Function on Juvenile Litopenaeus vannamei
Source: Antioxidants (Basel). 2023 Sep 27;12(10):1802. doi: 10.3390/antiox12101802 (PMC10604745; doi:10.3390/antiox12101802)
Supplement: Supplementary file 1 [file antioxidants-12-01802-s001.zip › antioxidants-2602816-SI.pdf]

Supplementary material

Evaluating the Antioxidant Properties of the Leaves and Stems of *Alpinia oxyphylla* In Vitro and Its Growth-Promoting, Muscle Composition Change, and Antioxidative Stress Function on Juvenile *Litopenaeus vannamei*

Jun-Tao Li [1,†], Yu-Hua Zhao [2,†], Yuan Lv [1,2,†], Xin Su [1], Wen-Li Mei [1], Yao-Peng Lu [1], Pei-Hua Zheng [1], Ze-Long Zhang [1], Xiu-Xia Zhang [1], Hui-Qin Chen [1,2,*], Hao-Fu Dai [1,*] and Jian-An Xian [1,*]

[1] Hainan Provincial Key Laboratory for Functional Components Research and Utilization of Marine Bio-Resources, Key Laboratory of Natural Products Research and Development from Li Folk Medicine of Hainan Province, Institute of Tropical Biosciences and Biotechnology, Chinese Academy of Tropical Agricultural Science, Key Laboratory for Biology and Genetic Resources of Tropical Crops of Hainan Province, Hainan Institute of Tropical Agricultural Resources, Haikou 571101, China; lijuntao@itbb.org.cn (J.-T.L.); 13006160035@163.com (Y.L.); suxinnpc@126.com (X.S.); meiwenli@itbb.org.cn (W.-L.M.); luyaopeng@itbb.org.cn (Y.-P.L.); zhengpeihua@itbb.org.cn (P.-H.Z.); zhangzelong@itbb.org.cn (Z.-L.Z.); zhangxiuxia@itbb.org.cn (X.-X.Z.)

[2] Key Lab of Freshwater Animal Breeding, Ministry of Agriculture, Key Lab of Agricultural Animal Genetics, Breeding and Reproduction of Ministry of Education, Freshwater Aquaculture Collaborative Innovation Center of Hubei Province, College of Fisheries, Huazhong Agricultural University, Wuhan 430070, China; zhaoyuhua2005@mail.hzau.edu.cn

* Correspondence: chenhuiqin@itbb.org.cn (H.-Q.C.); daihaofu@itbb.org.cn (H.-F.D.); xianjianan@itbb.org.cn (J.-A.X.)

† These authors contributed equally to this work.

Contents

1. **Table S1** Primer information of Real-time Fluorescence Quantitative PCR.........3

2. ^{1}H and ^{13}C NMR, and HEESIMS spectra of compounds **1** and **2**

Figure S1 ^{1}H NMR spectrum of compound **1** in MeOD.............................4

Figure S2 ^{13}C NMR spectrum of compound **1** in MeOD.............................4

Figure S3 HRESIMS spectrum of compound **1**..5

Figure S4 ^{1}H NMR spectrum of compound **2** in MeOD.............................6

Figure S5 ^{13}C NMR spectrum of compound **2** in MeOD..........................6

Figure S6 HRESIMS spectrum of compound **2**..7

Table S1 Primer information of Real-time Fluorescence Quantitative PCR

Genes	Sequence No.	Primer sequences (5′-3′)
β-actin-F	AF300705	GCCCATCTACGAGGGATA
β-actin-R		GGTGGTCGTGAAGGTGTAG
proPo-F	AY723296.1	TCCATTCCGTCCGTCTG
proPo-R		GGCTTCGCTCTGGTTAGG
Mn-SOD-F	DQ005531	CTGGTTCCGTTGCTTGGC
Mn-SOD-R		CGCTCATTCACGTTCTCCC
CAT-F	AY518322	TCAGCGTTTGGTGGAGAA
CAT-R		GCCTGGCTCATCTTTATC
GSH-PX-F	AY973252	AGGGACTTCCACCAGATG
GSH-PX-R		CAACAACTCCCCTTCGGTA

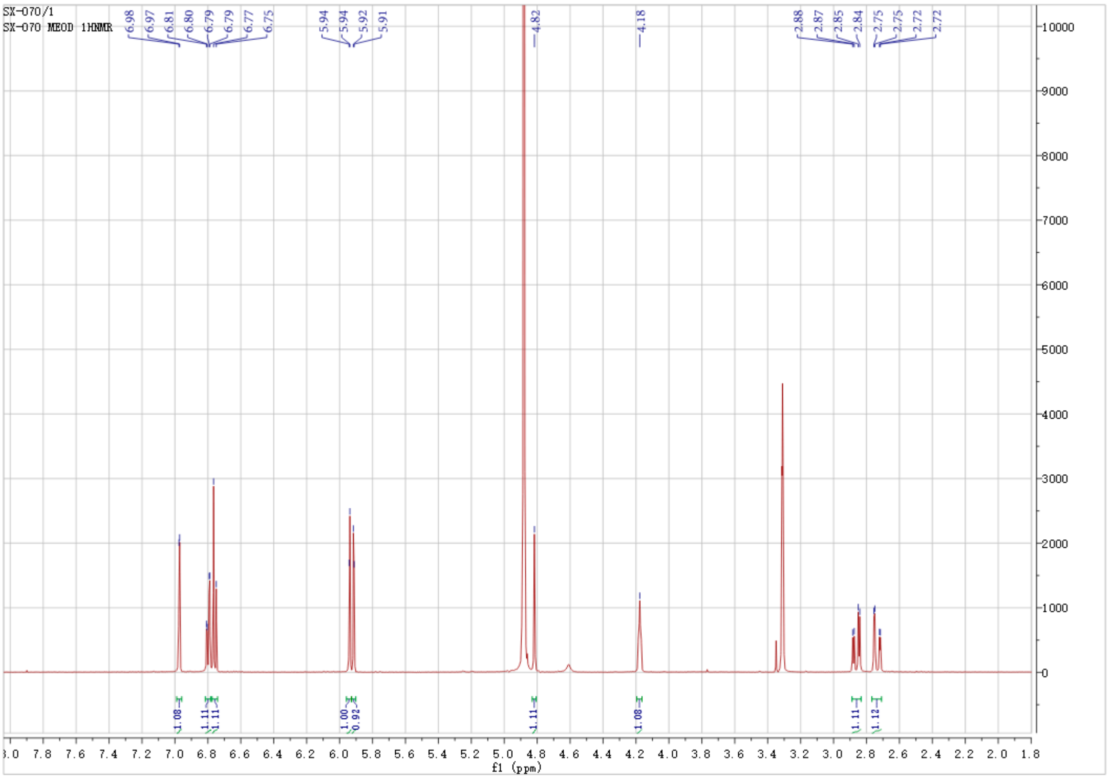

Figure S1 ^{1}H NMR spectrum of compound **1** in MeOD

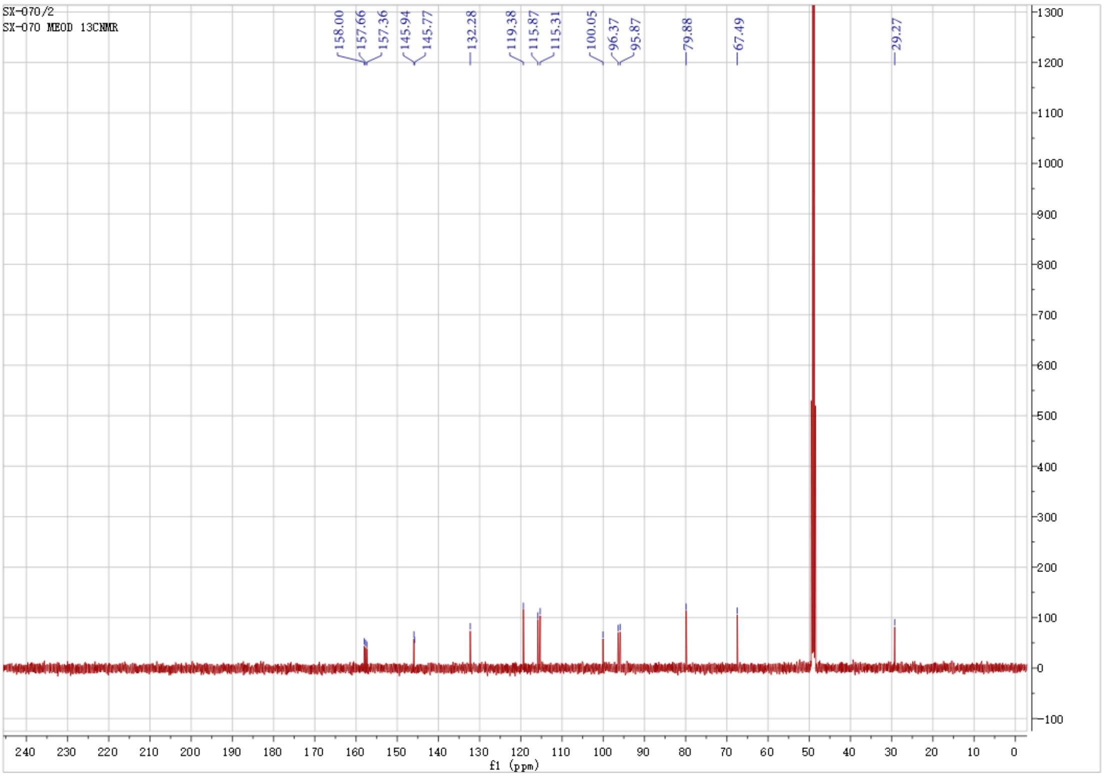

Figure S2 ^{13}C NMR spectrum of compound **1** in MeOD

Mass Spectrum SmartFormula Report

Analysis Info

Analysis Name	D:\2023\A501\WYL\20230718\WF-comp.1_GA1_01_7984.d	Acquisition Date	2023-07-18 09:49:42
Method	10%M-suanshui-160min.m	Operator	Demo User
Sample Name	WF-comp.1	Instrument	compact 8255754.20156
Comment			

Acquisition Parameter

Source Type	ESI	Ion Polarity	Positive	Set Nebulizer	1.8 Bar
Focus	Not active	Set Capillary	3500 V	Set Dry Heater	220 °C
Scan Begin	50 m/z	Set End Plate Offset	-500 V	Set Dry Gas	4.0 l/min
Scan End	2500 m/z	Set Charging Voltage	2000 V	Set Divert Valve	Waste
		Set Corona	0 nA	Set APCI Heater	0 °C

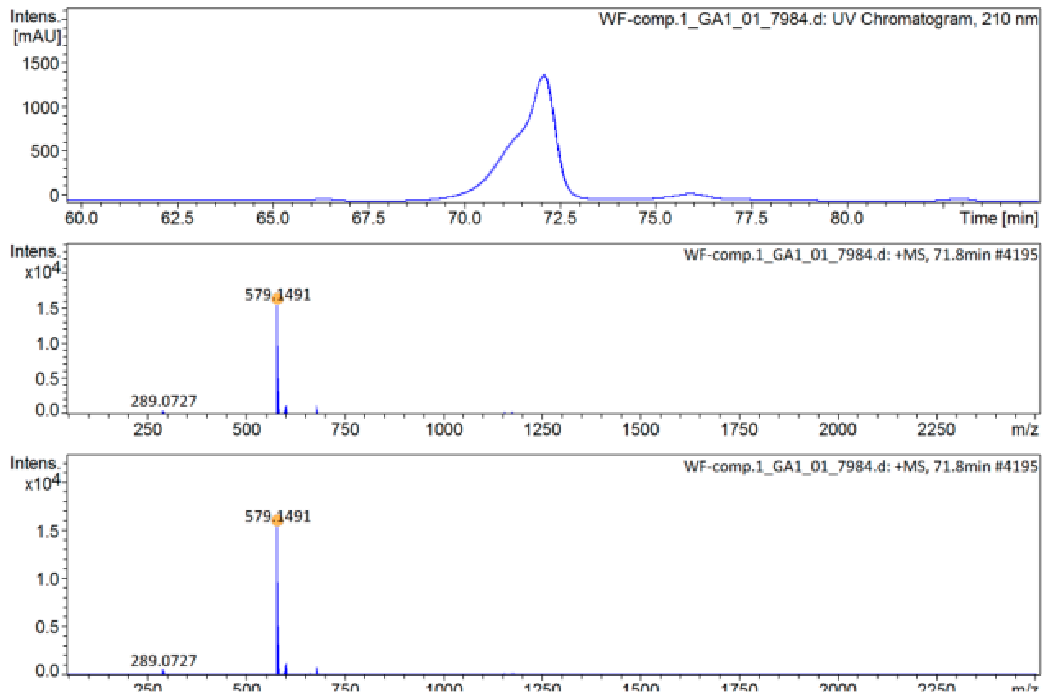

Meas. m/z	#	Ion Formula	m/z	err [ppm]	mSigma	# mSigma	Score	rdb	e⁻ Conf	N-Rule	Adduct
579.1491	1	C30H27O12	579.1497	1.1	2.5	1	100.00	18.0	even	ok	M+H

Figure S3 HRESIMS spectrum of compound **1**

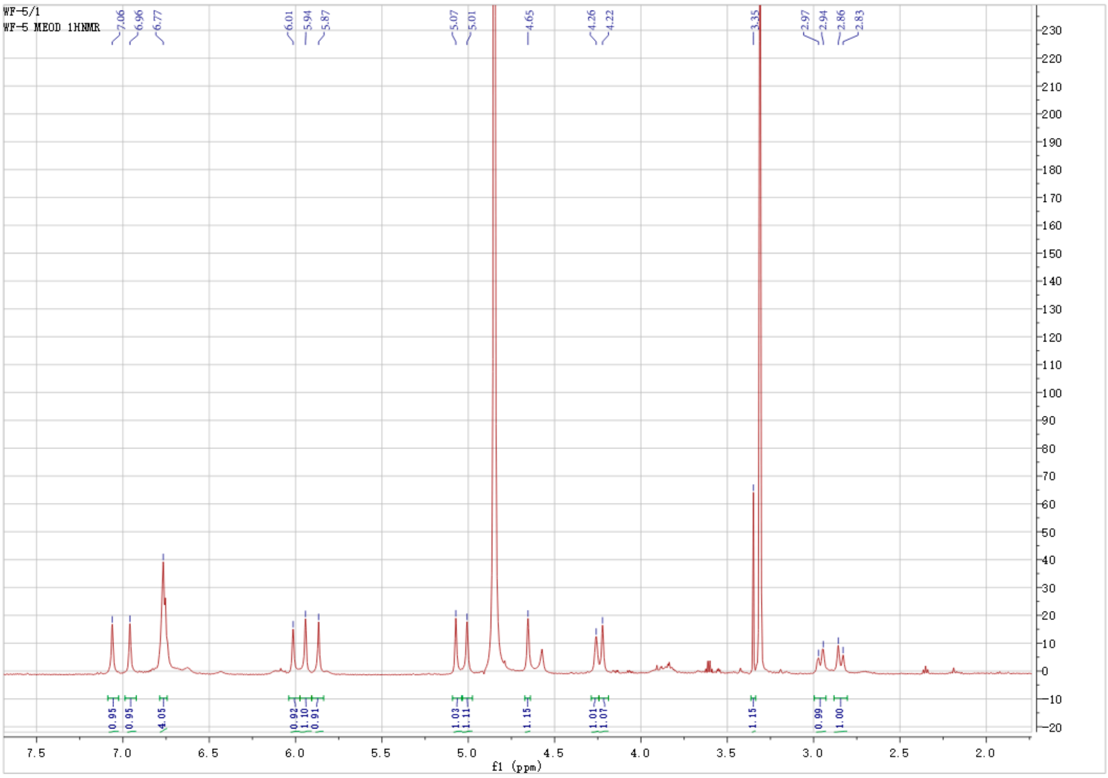

Figure S4 ^{1}H NMR spectrum of compound **2** in MeOD

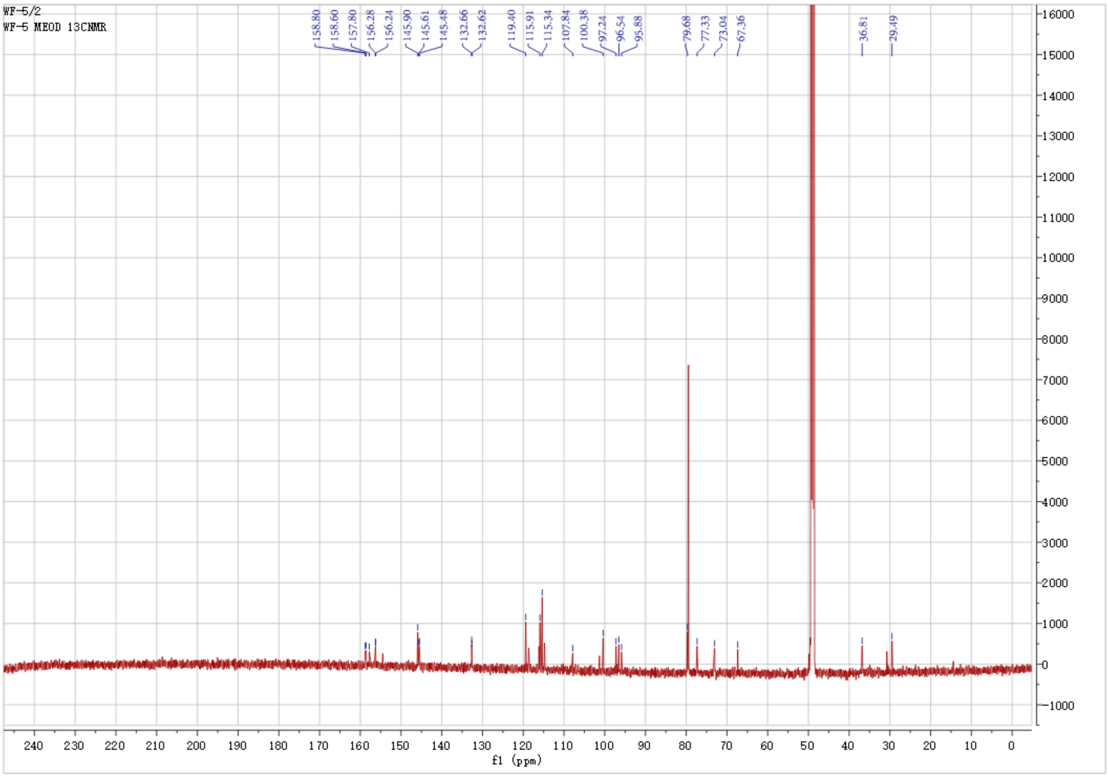

Figure S5 ^{13}C NMR spectrum of compound **2** in MeOD

Mass Spectrum SmartFormula Report

Analysis Info

Analysis Name	D:\2023\A501\WYL\20230718\WF-comp.2_GA2_01_7985.d	Acquisition Date	2023-07-18 12:32:09
Method	10%M-suanshui-160min.m	Operator	Demo User
Sample Name	WF-comp.2	Instrument	compact 8255754.20156
Comment			

Acquisition Parameter

Source Type	ESI	Ion Polarity	Positive	Set Nebulizer	1.8 Bar
Focus	Not active	Set Capillary	3500 V	Set Dry Heater	220 °C
Scan Begin	50 m/z	Set End Plate Offset	-500 V	Set Dry Gas	4.0 l/min
Scan End	2500 m/z	Set Charging Voltage	2000 V	Set Divert Valve	Waste
		Set Corona	0 nA	Set APCI Heater	0 °C

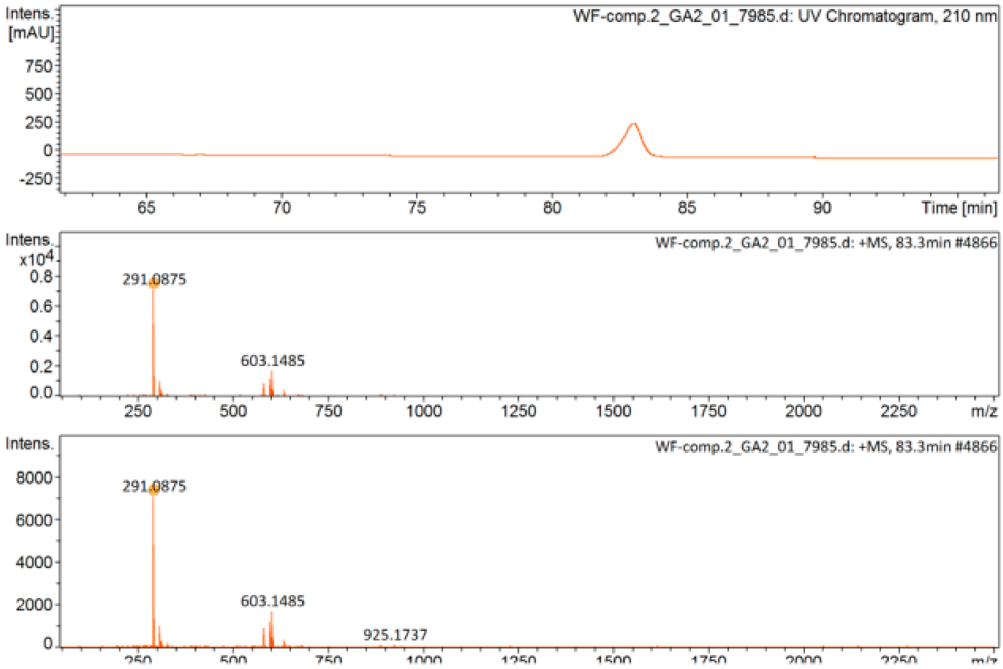

Meas. m/z	#	Ion Formula	m/z	err [ppm]	mSigma	# mSigma	Score	rdb	e¯ Conf	N-Rule	Adduct
291.0875	1	C15H15O6	291.0863	-4.1	15.4	1	100.00	9.0	even	ok	M+H

Figure S6 HRESIMS spectrum of compound **2**
